# The Book World of Medicine and Science

**Published:** 1904-08-13

**Authors:** 


### The Book World of Medicine and Science.

International Clinics. Vol. II. Fourteenth Series. (London: Lippincott Company. Pp. 314. With illustrations. Price 10s. 6d.)

Although on many previous occasions we have had the pleasure of remarking upon the excellence of the successive volumes of "International Clinics," we do not think the present volume has been surpassed by any of its predecessors. The chief feature is the section on Diseases of Warm Climates, which occupies nearly half the book, and contains papers on the spread of diseases by insects with suggestions regarding prophylaxis, recent progress in tropical medicine, sleeping sickness, etiology of bilious hæmoglobinuric fever, malarial hæmoglobinuria, uncinariasis, liver abscess, its diagnosis and treatment, and the treatment and mode of life to be pursued on return to a cold climate by those suffering from the commoner affections incidental to a sojourn in tropical countries. The subject matter of these papers has such a lively interest at the present time that they are likely to attract a greater share of attention than will the sections on treatment, medicine, surgery, pediatrics, and rhinology, which, however, contain several exceptionally good articles, while a high standard of excellence is maintained throughout. These "International Clinics" should find a place in every medical library.

An Index of Symptoms as a Clue to Diagnosis. By Ralph Winnington Leftwich, M.D. Third edition. (London: Smith, Elder and Co. 1904. Pp. 399. Price 6s. net.)

The appearance of a third edition of Dr. Leftwich's manual is convincing evidence of its value. The plan of the book, as doubtless many of our readers know, is the presentation under each of the symptoms or clinical evidences of disease of its several possible causes. Hence the practitioner, puzzled by some condition for which he can find no confident explanation, may turn to this "Index of Symptoms" with a view to revise his knowledge of the various circumstances in which this condition arises. The book, in short, is a guide from symptoms to diagnosis—the reverse, that is, of the ordinary systematic text-book, in which each disease is first of all named, and then its symptoms are described. But something more than mere lists of possible diagnoses are included in Dr. Leftwich's book. An attempt is made to distinguish between probable and remotely possible causes of the various symptoms, and many helpful hints are given in the direction of accurate diagnosis and the avoidance of easily committed errors. Necessarily, a scheme such as this has its limitations, but, if wisely employed, it can claim without doubt an area of definite value in the art of practical medicine. On points of detail there may even yet be room for revision. Thus it is surely incorrect to say that the murmur of mitral stenosis is "usually soft"; the illustration on page 238 is wrongly named; and the figures of pulse tracings are far from successful. A special word of praise is due to the third edition for the completeness which marks its recognition of new terms and symptoms. All the latest clinical observations, many of which are known by the names of those who first described them, are here named and defined. Thoroughness, indeed, is a feature of the book.

The Prevention of Disease in Armies in the Field. By Robert Caldwell, F.R.C.S., D.P.H., Major R.A.M.C. (Baillière, Tindall and Cox. 1904. Pp. 178. Price 5s. net.)

There is a special force and charm attached to this book, arising from the fact that it is mainly a record of personal experiences. The author has been brought face to face with the difficulties and problems of military and camp sanitation, and he writes of these in a thoughtful and practical spirit. Of the importance of the subject there is no question. The disease mortality of a campaign is invariably much more serious than that arising from the bullets of the enemy; and, apart from the question of deaths by disease, there is the influence of the same factor on the physique and efficiency of the troops. We consider Major Caldwell to have made a wise and effective use of his experience, and his recommendations are worthy of the most careful consideration by those in authority. His book is an extremely interesting and lucid account of a medical officer's personal observations in the South African war, and we hope it will attain a wide circulation.

Acid Auto-intoxications. By Professor Dr. Carl von Noorden and Dr. Mohr. Authorised Translation under the direction of Boardman Reed, M.D. (Bristol: Jno. Wright and Co.; London: Simpkin, Marshall, Hamilton, Kent and Co. 1904. Pp. 80.)

This is one of a series of clinical treatises on the pathology and therapy of disorders of metabolism and nutrition by Professor von Noorden. The present essay deals with the question of auto-intoxication by the acid products of metabolism, and is marked by the scientific method which characterises the clinical work of its distinguished author. There is in addition abundance of practical application, more especially in the dietetic and medicinal treatment of diabetes. The importance of the study of digestion, assimilation, and excretion in patients who are the subjects of chronic diseases can hardly be exaggerated, and von Noorden's work is a valuable contribution in this direction.

Emergency Pocket Case-Book. Designed by a Captain in the R.A.M.C. (Reynolds and Branson, Ltd., Leeds. Price 6d.)

To all students and practitioners who keep notes of their cases this little pocket-book of charts is likely to prove useful. It is sufficiently small to carry in the coat pocket, and contains 30 charts with spaces for noting the patient's name and condition on admission, his temperature, pulse, and respiration, the medicine and diet ordered, and the daily progress of the case. In fact all the most important pathological details can be recorded from day to day by the bedside with a minimum of trouble, and preserved for future reference.

"Red Book" on Advertising. (London: F. E. Coe, 19–21 High Holborn, W.C. Price 2s. 6d. net.)

This book contains lists of the leading newspapers of the United Kingdom, the Continent of Europe and America, together with information concerning each publication of practical value to advertisers, given very clearly and concisely, while the interesting articles on advertising should certainly prove useful to the novice who is anxious for success and does not know exactly how to attain it. We think the value of future editions of the book would be greatly enhanced by the addition of the principal Colonial journals, a point which has apparently escaped the attention of the compilers on this occasion.

